# Left and right ventricular global longitudinal strain assessment together with biomarker evaluation may have a predictive and prognostic role in patients qualified for hematopoietic stem cell transplantation due to hematopoietic and lymphoid malignancies – a pilot study description

**DOI:** 10.1186/s40959-024-00210-8

**Published:** 2024-02-17

**Authors:** Bartosz Puła, Jarosław Kępski, Irena Misiewicz-Krzemińska, Sebastian Szmit

**Affiliations:** 1 Department of Hematology, Institute of Hematology and Transfusion Medicine, Warsaw, Poland; 2 Department of Cardio-Oncology, Chair of Hematology and Transfusion Medicine, Centre of Postgraduate Medical Education, Warsaw, Poland; 3 Department of Experimental Hematology, Institute of Hematology and Transfusion Medicine, Warsaw, Poland

**Keywords:** Hematopoietic stem cell transplantation, Global longitudinal strain, Echocardiography, Cardio-oncology, Hemato-oncology

## Abstract

The hematopoietic stem cell transplantation (HSCT) procedure is considered a cardiovascular burden. This is due to the potentially cardiotoxic cytostatic agents used before and the risks associated with peri-transplant procedures. We designed a pilot study to determine the clinical utility of the new ST2 marker; furthermore, we routinely assessed cardiac parameters in HSCT recipients. Based on previous cardio-oncology experience in lung and prostate cancer, we can confirm the prognostic and predictive value of classic cardiac biomarkers and modern echocardiography parameters such as global longitudinal strain of the left and right ventricle. After conducting this pilot study we can create a predictive and prognostic model for patients undergoing HSCT. This will greatly enrich our clinical practice, especially in treating older people.

## Introduction

The procedure of bone marrow transplantation (HSCT, *hematopoietic stem cell transplantation*), due to cytostatic agents used and the risks associated with peri-transplant procedures, is considered a cardiovascular burden. Therefore, assessing the cardiopulmonary fitness and risk factors for cardiovascular complications of patients qualified for this procedure is important. The most recent European Society of Cardiology (ESC) recommendations on cardio-oncology from August 2022, prepared jointly with the European Hematology Association, recommend echocardiography in patients before HSCT, but there is no information as to what echocardiographic parameters may have prognostic significance in this specific patient population [1]. An earlier expert document published in 2020 by the Cardio-Oncology Study Group of the Heart Failure Association of the European Society of Cardiology highlighted the importance of baseline cardio-oncologic risk stratification and described extensively the aspects of anticancer therapy but completely omitted the issue of patients qualified for HSCT [2].

The European Society of Cardiology’s recommendations on heart failure, published in August 2021, presents a list of anticancer drugs that can cause heart failure [3]. Several of these drugs are used in hematology, including, but not limited to, in patients undergoing a subsequent bone marrow transplant procedure. Earlier in 2020, the results of an international European registry (CARDIOTOX registry) had been published, which showed a significant association between the diagnosis of severe cardiotoxicity of anticancer drugs and the risk of premature death and significantly shorter overall survival [4]. The next part of the registry revealed that the co-occurrence of classic risk factors (e.g., hypertension, diabetes, older age) has additional negative implications for the prognosis of these patients [5]. A new definition of cardiotoxicity proposed by the International Cardio-Oncology Society was published on-line in December 2021 [6]. For the first time, an attempt was made to standardize diagnoses for the degree of myocardial damage caused by oncology or hematology drugs. The document attempts to reach a consensus on the positions of various cardiovascular societies (the European and American ones); moreover, it refers to the existing evaluation criteria in oncology clinical trials proposed by the US National Cancer Institute (Common Terminology Criteria for Adverse Events, CTCAE). The new proposed diagnostics are largely based on left ventricular ejection fraction (LVEF) assessment and global longitudinal strain (GLS) analysis of the left ventricle. Currently, the role of GLS assessment in patients undergoing HSCT is unknown.

In 2020, various ESC expert groups also proposed rules for echocardiographic and biomarker-based monitoring of patients receiving potentially cardiotoxic anticancer treatment [7, 8]. However, the monitoring rules in these documents were not dedicated to patients undergoing HSCT.

The determination of troponins and N-terminal fragment (pro) of B-type natriuretic peptide (NT-proBNP) is commonly used in cardiovascular risk assessment, but both markers have their limitations. A useful new marker in cardio-oncology may be the ST2 protein. The *IL1RL1* gene encodes ST. So far, it has been shown to be involved in the immune response. An increase in its concentration is observed in response to myocardial stretch (which may be relevant in various situations related to infectious complications we observe after bone marrow transplantation). Data published to date indicate that high ST2 levels may correlate with adverse outcomes in myocardial infarction, acute coronary syndrome, and worsening heart failure [9–11]. The unfavorable significance of high ST2 levels before the HSCT procedure has been demonstrated in the pediatric population [12]. Thus, ST2 may have predictive and clinical relevance in the qualification of patients for HSCT as an additional parameter to the risk parameters recognized so far, i.e. age, HCT-CI risk score, and intensity of conditioning, as well as new potential markers to optimize the outcomes of this procedure [13–16].

## Research hypothesis

Changes in left and right ventricular GLS assessment and biomarker concentrations may reflect even subclinical cardiovascular damage and play both a predictive and prognostic role during anticancer treatment and HSCT procedures.

High ST2 and mediators of vascular inflammation levels could correlate with the degree of myocardial damage during anticancer treatment and HSCT procedures. In addition, high ST2 levels in the serum of patients undergoing HSCT may constitute an adverse immune-related factor during the HSCT procedure.

### Study design

**Fig. 1:**
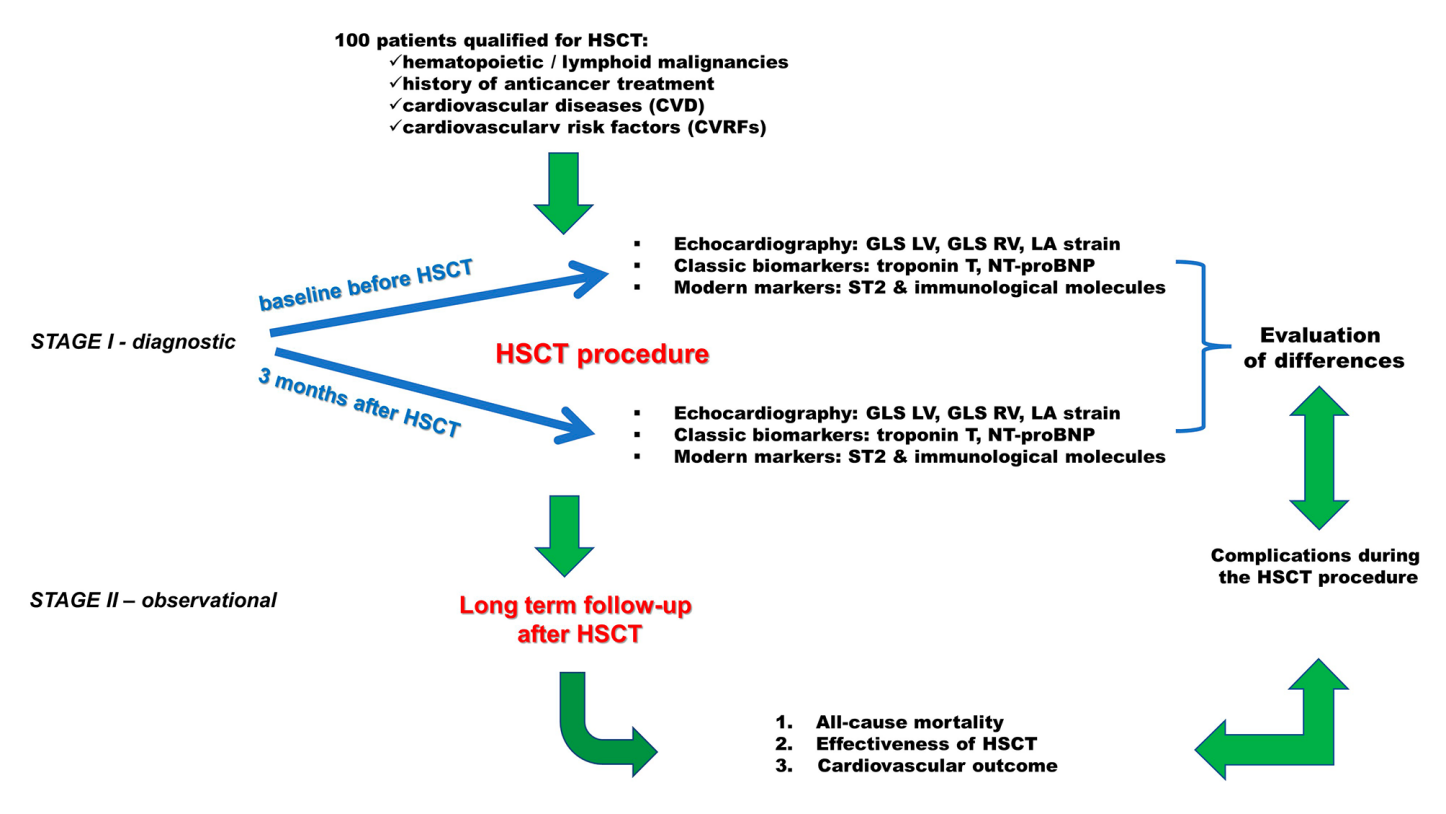
Design of the study

In order to confirm the research hypothesis we designed a pilot study to determine the clinical utility of modern echocardiography, classic cardiac biomarkers, and the new ST2 marker among others immunological markers. The study will be carried out in two stages (Fig. 1):

#### STAGE I – diagnostic

Cardiac echocardiography with assessment of left and right ventricular GLS, left atrial strain, and determination of biomarkers (troponin T, NT-proBNP, ST2) will be performed in patients with a diagnosis of hematologic malignancy (1) as part of the qualification for the bone marrow transplant procedure and (2) three months after HSCT. The study will include one hundred patients in the project’s first two years. Patient material (serum and EDTA plasma) will be banked consecutively.

The design of the study makes it possible that the samples are shipped from other centers to the central laboratory within 24 h at refrigerating conditions. The biomarker determinations (including troponin T, NT-proBNP and ST2) will be performed by ELISA and capillary nanoimmunoelectrophoresis (CNIA) using a specific antibody against ST2, among others, as the material is obtained. As a reference, level of troponin-T and NT-proBNP will be determined in a medical diagnostic laboratory using certified IVD procedures. For CNIA evaluation, immunoglobulins and albumin will be removed from the serum portions, followed by samples labeled with fluorescent markers and separated on a matrix in capillaries, where the specific determination of the protein under study will also occur. Moreover, using flow cytometry and LEGENDplex™ Human Vascular Inflammation Panel 2, the following molecules will be quantified: sST2, sRAGE, TIE-2, sCD40L, TIE-1, sFlt-1, LIGHT, TNF-α, PlGF, IL-6, IL-18, IL-10, CCL2 (MCP-1).

#### STAGE II – observational

The results of tests performed at baseline will be correlated with the incidence of complications during the HSCT procedure. Additionally, results of tests performed before the HSCT procedure will be compared with findings in echocardiography and biomarkers levels after the HSCT procedure, then correlated with the type of prior anticancer treatment and finally related to the prognosis of patients (effectiveness of bone marrow transplantation, cardiovascular complications, subsequent hospitalizations for any cause including cardiac hospitalizations, cardiovascular and all-cause mortality).

### Clinical utility

The implementation of this two-stage design should allow for fulfilling the objectives of the project:

(1) To determine the relevance of the diagnosis of left and right ventricular GLS abnormalities and selected biomarkers (including troponin T, NT-proBNP and ST2 and other relevant proteins included in the panel) to the clinical course of HSCT; (2) To determine the relationship between the type of prior hemato-oncologic treatment and the risk of severe, moderate and mild CTRCD (cancer therapy-related cardiac dysfunction) in patients qualified for HSCT; (3) Identification of echocardiographic criteria and changes in the concentration of the above biomarkers, allowing early identification of patients at risk of cardiovascular complications of the HSCT procedure, including subclinical myocardial damage, vascular disorders (thromboembolic events, hypertension etc.) and different types of arrhythmias.

The possibility of samples shipment to the central laboratory where the biomarkers will be assessed opens an opportunity for this pilot experiment to evolve into a multicenter study. We will consider doing so after obtaining strong preliminary data from the present pilot study.

### Characteristics of the first 30 enrolled patients

It was originally assumed that the study would be a single-center experience, conducted in the Polish reference hospital for hematology. The basic goal was to test the initial hypothesis, i.e. that modern echocardiographic parameters can play a predictive and prognostic role in HSCT. The clinical characteristics of patients before HSCT have been presented in Table 1.

**Table 1:** Clinical characteristics of the first 30 patients with hematological malignancies before HSCT included in the study

		Number of patients	(%)
Sex	Female Male	14 16	(46.67%) (53.33%)
Age (years)	Mean: 49.1 ± 14.2 Min-max: 21 to 71		
	over 60 years old	8	(26.67%)
Hematological diagnoses	Acute myeloid leukaemia Multiple myeloma Acute lymphoblastic leukaemia Myelodysplastic syndromes Non-Hodgkin lymphoma Chronic myeloid leukaemia Myelofibrosis Amyloidosis	10 9 3 3 2 1 1 1	(33.33%) (30%) (10%) (10%) (6.67%) (3.33%) (3.33%) (3.33%)
History of cardiovascular disorders	Arterial hypertension Atrial fibrillation Coronary artery disease Heart failure	6 1 1 1	(20%) (3.33%) (3.33%) (3.33%)

The clinical cardiovascular risk profile observed amongst the included patients seems to be prognostically more beneficial than the risk profile observed amongst patients with newly diagnosed hematological malignancies [17]. Patients with concomitant cardiovascular diseases or even only risk factors for these diseases are less likely to be considered for HSCT. That is further reason why prognostic markers should be sought amongst sensitive echocardiographic parameters and biomarkers as they can identify patients even with subclinical myocardial damage, regardless of the ischemic or toxic or immune-related etiology.

The echocardiographic characteristics of included patients before HSCT were presented in Table 2. It should be noticed that in 7 patients there is no LV GLS and in 9 patients there is no RV GLS assessment, mainly due to quality of visibility. In 14 patients there is no LA GLS; this is mainly due to insufficient visibility and very high variability of the parameter.

**Table 2:** Baseline echocardiography characteristics of 30 included patients

Parameter	Median	Min. to max. value
Left ventricular ejection fraction (EF, %)	62	50 to 68
Left Atrial Volume Index (LAVI, ml/m2)	32	18 to 54
Tricuspid regurgitation velocity (TRV, m/s)	2.3	1.8 to 2.8
E/e’ ratio	6.5	3.5 to 10
Tricuspid annular plane systolic excursion (TAPSE, mm)	22	14 to 30
Peak S wave velocity of the lateral tricuspid annulus by tissue Doppler imaging(RVS’, cm/s)	12	9.3 to 19
Left ventricular global longitudinal strain* (LV GLS, %)	− 19.4	− 24.0 to -16.5
Right vetricular global longitudinal strain** (RV GLS, %)	-24	− 30 to − 15
Left atrial global longitudinal strain*** (LA GLS, %)	39	27 to 50

*estimated in 23 patients / **estimated in 21 patients / ***estimated in 16 patients

A shortfall of the current stage of the study was the fact that amongst included patients there were some cases in whom a satisfactory image of the left ventricular endocardium was not obtained. This prevented reliable assessment of myocardium strain. The main reason for this study limitation was obesity or tachycardia. Nevertheless, we plan to attempt to evaluate GLS in the following stages of our study. If the preliminary results confirm that the assessed modern echocardiographic parameters can play a role in predicting short- and long-term outcome in HSCT, the next phase of the study will involve multi-center cooperation. The Echocardiography Laboratory at the Institute of Hematology and Transfusion Medicine in Warsaw will remain the central CORE ECHO LAB. One of the reasons is the fact that said Laboratory is a unique Polish center that has the status of Cardio-Oncology Center of Excellence with the highest designation GOLD confirmed by the International Cardio-Oncology Society.

## Conclusion

Based on previous cardio-oncology experience in lung and prostate cancer, we can confirm the prognostic and predictive value of classic cardiac biomarkers (D-dimer, NT-proBNP, troponin), modern biomarkers, and echocardiography parameters such as GLS of the left and right ventricle [18–21]. After conducting this pilot study, we can create a predictive and prognostic model for patients undergoing HSCT. This will greatly enrich our clinical practice, especially in treating older people.

## Data Availability

All data of the study will be available upon reasonable request and with permission of the Directors of the Institute of Hematology and Transfusion Medicine in Warsaw, Poland.
